# Supplementary material for: Intensive community and home-based treatments for eating disorders: a scoping review study protocol
Source: BMJ Open. 2023 Feb 15;13(2):e064243. doi: 10.1136/bmjopen-2022-064243 (PMC9933757; doi:10.1136/bmjopen-2022-064243)
Supplement: Supplementary data [file bmjopen-2022-064243supp001.pdf]

Supplemental material

This document provides details of the pilot search conducted on the main databases on 01/07/2022.

Pilot search on PubMed

	Query	Results
**#1**	"Home Care Services"[Mesh] OR "Home Care Services, Hospital-Based"[Mesh] OR "Community Mental Health Services"[Mesh] OR "Community Mental Health Centers"[Mesh]	71545
**#2**	"community care*"[Title/Abstract] OR "community treat*"[Title/Abstract] OR "intensive community*"[Title/Abstract] OR "intensive outpatient*"[Title/Abstract] OR "home care*"[Title/Abstract] OR "home treat*"[Title/Abstract] OR "home feed*"[Title/Abstract]	31346
**#3**	"Anorexia Nervosa"[MeSH Terms] OR "Anorexia"[MeSH Terms] OR "Avoidant Restrictive Food Intake Disorder"[MeSH Terms] OR "Binge-Eating Disorder"[MeSH Terms] OR "Bulimia Nervosa"[MeSH Terms] OR "Bulimia"[MeSH Terms] OR"Feeding and Eating Disorders"[MeSH Terms]	41989
**#4**	"eating disorder*"[Title/Abstract] OR "anorex*"[Title/Abstract] OR "bulimi*"[Title/Abstract] OR "binge eating*"[Title/Abstract] OR "Avoidant Restrictive Food Intake Disorder" OR"ARFID"[Title/Abstract]	57264
**#5**	#1 OR #2	88293
**#6**	#3 OR #4	68288
**#7**	#5 AND #6	238

Pilot search on APA PsycInfo

	Query	Results
**#1**	exp Community Mental Health Services/ or exp Community Mental Health Centers/	10681
**#2**	exp Home Care/	7190
**#3**	(community care* or community treat* or intensive community* or intensive outpatient*).mp.	6807
**#4**	(home adj3 care*).mp.	19244
**#5**	(home adj3 treat*).mp.	1927
**#6**	(home adj3 feed*).mp.	178
**#7**	exp Eating Disorders/	33619
**#8**	(Feeding and Eating Disorder*).mp.	9239
**#9**	(anorex* or bulimi* or binge eat* or Avoidant Restrictive Food Intake Disorder or ARFID).mp.	29770
**#10**	(eating adj3 disorder*).mp.	35442
**#11**	#1 or #2 or #3 or #4 or #5 or #6	36110
**#12**	#7 or #8 or #9 or #10	49186
**#13**	#11 and #12	204

æ6R, *et al. BMJ Open* 2023; 13:e064243. doi: 10.1136/bmjopen-2022-064243

Pilot search on Ovid MEDLINE

	Query	Results
**#1**	exp Community Mental Health Centers/ or exp Community Mental Health Services/	21939
**#2**	exp Home Care Services/	50063
**#3**	(community care* or community treat* or intensive community* or intensive outpatient*).mp. [mp=title, abstract, original title, name of substance word, subject heading word, floating sub-heading word, keyword heading word, organism supplementary concept word, protocol supplementary concept word, rare disease supplementary concept word, unique identifier, synonyms]	8610
**#4**	(home adj3 care*).mp. [mp=title, abstract, original title, name of substance word, subject heading word, floating sub-heading word, keyword heading word, organism supplementary concept word, protocol supplementary concept word, rare disease supplementary concept word, unique identifier, synonyms]	65503
**#5**	(home adj3 treat*).mp. [mp=title, abstract, original title, name of substance word, subject heading word, floating sub-heading word, keyword heading word, organism supplementary concept word, protocol supplementary concept word, rare disease supplementary concept word, unique identifier, synonyms]	5993
**#6**	(home adj3 feed*).mp. [mp=title, abstract, original title, name of substance word, subject heading word, floating sub-heading word, keyword heading word, organism supplementary concept word, protocol supplementary concept word, rare disease supplementary concept word, unique identifier, synonyms]	670
**#7**	exp "Feeding and Eating Disorders"/	34639
**#8**	(anorex* or bulimi* or binge eating* or Avoidant Restrictive Food Intake Disorder or ARFID).mp. [mp=title, abstract, original title, name of substance word, subject heading word, floating sub-heading word, keyword heading word, organism supplementary concept word, protocol supplementary concept word, rare disease supplementary concept word, unique identifier, synonyms]	50023
**#9**	(eating adj3 disorder*).mp. [mp=title, abstract, original title, name of substance word, subject heading word, floating sub-heading word, keyword heading word, organism supplementary concept word, protocol supplementary concept word, rare disease supplementary concept word, unique identifier, synonyms]	32939
**#10**	#1 or #2 or #3 or #4 or #5 or #6	107933
**#11**	#7 or #8 or #9	69172
**#12**	#10 and #11	293

æ6R, *et al. BMJ Open* 2023; 13:e064243. doi: 10.1136/bmjopen-2022-064243

Pilot search on Web of Science

	Query	Results
**#1**	TS=("eating disorder*" OR "anorex*" OR "bulimi*" OR "binge eat*" OR "Avoidant Restrictive Food Intake Disorder" OR "ARFID")	70790
**#2**	TS=((community NEAR/3 "care*”) OR (community NEAR/3 "treat*”) OR (intensive NEAR/3 "community*”) OR (intensive NEAR/3 "outpatient*”) OR (home NEAR/3 "care*”) OR (home NEAR/3 "treat*”) OR (home NEAR/3 " feed*”))	99666
**#3**	#1 AND #2	288

æ6R, *et al. BMJ Open* 2023; 13:e064243. doi: 10.1136/bmjopen-2022-064243
