# Supplementary material for: Intensive community and home-based treatments for eating disorders: a scoping review study protocol
Source: BMJ Open. 2023 Feb 15;13(2):e064243. doi: 10.1136/bmjopen-2022-064243 (PMC9933757; doi:10.1136/bmjopen-2022-064243)
Supplement: Author's manuscript [file bmjopen-2022-064243.draft_revisions.pdf]

BMJ Open is committed to open peer review. As part of this commitment we make the peer review history of every article we publish publicly available.

When an article is published we post the peer reviewers' comments and the authors' responses online. We also post the versions of the paper that were used during peer review. These are the versions that the peer review comments apply to.

The versions of the paper that follow are the versions that were submitted during the peer review process. They are not the versions of record or the final published versions. They should not be cited or distributed as the published version of this manuscript.

BMJ Open is an open access journal and the full, final, typeset and author-corrected version of record of the manuscript is available on our site with no access controls, subscription charges or pay-per-view fees (http://bmjopen.bmj.com).

If you have any questions on BMJ Open's open peer review process please email info.bmjopen@bmj.com

Intensive Community and Home-Based Treatments for Eating Disorders: A Scoping Review Study Protocol

Journal:	*BMJ Open*
Manuscript ID	bmjopen-2022-064243
Article Type:	Protocol
Date Submitted by the Author:	26-Apr-2022
Complete List of Authors:	İnce, Başak; King's College London Institute of Psychiatry Psychology and Neuroscience, Department of Psychological Medicine - Section of Eating Disorders<br>Phillips, Matthew; King's College London Institute of Psychiatry Psychology and Neuroscience, Department of Psychological Medicine - Section of Eating Disorders<br>Schmidt, Ulrike; King's College London Institute of Psychiatry Psychology and Neuroscience, Department of Psychological Medicine - Section of Eating Disorders; South London and Maudsley NHS Foundation Trust, Eating Disorder Outpatients Service
Keywords:	Eating disorders < PSYCHIATRY, Adult psychiatry < PSYCHIATRY, Child & adolescent psychiatry < PSYCHIATRY, PUBLIC HEALTH

SCHOLARONE™
Manuscripts

Intensive Community and Home-Based Treatments for Eating Disorders: A Scoping Review Study Protocol

Başak İnce [1], Matthew Phillips [1], Ulrike Schmidt [1,2]

[1] Department of Psychological Medicine, Section of Eating Disorders, Institute of Psychiatry, Psychology & Neuroscience, King's College London, London, UK

[2] Eating Disorder Outpatients Service, South London and Maudsley NHS Foundation Trust, London, UK

Corresponding author: Başak İnce (basak.ince@kcl.ac.uk)

Department of Psychological Medicine, Section of Eating Disorders, Institute of Psychiatry, Psychology & Neuroscience, King's College London, London SE5 8AF, UK.

ORCID:

Başak İnce: http://orcid.org/0000-0003-1177-3490

Matthew Phillips: https://orcid.org/0000-0001-8250-5560

Ulrike Schmidt: http://orcid.org/0000-0003-1335-1937

For peer review only

Word count: 2790

For peer review only - http://bmjopen.bmj.com/site/about/guidelines.xhtml

ABSTRACT

Introduction: Institutionally based intensive treatment modalities (inpatient, day patient, and residential treatments) for eating disorders (ED) are associated with high treatment costs and significant challenges for patients and carers, including access difficulties and disruption to daily routines. Intensive community and home-based treatments have been suggested as alternatives to institutionally based intensive treatments for other severe mental illnesses, with promising clinical, social, and health economic outcomes. The possible advantages of these treatments have been proposed for EDs, but this emerging area of research has not yet been systematically investigated. This scoping review aims to map the available literature on intensive community and home treatments for EDs, focusing on their conceptualisation, implementation, and clinical outcomes.

Methods: This proposed scoping review will follow the Preferred Reporting Items for Systematic Review and Meta-Analysis Protocol extension for Scoping Reviews checklist and the Joanna Briggs Institute Reviewer's Manual. This review will include any peer-reviewed study concerning intensive community and home-based treatments for any EDs, with no restrictions on geographical context or study design. Grey literature will also be considered. The literature search will be conducted in four databases: PubMed, PsycInfo, MEDLINE, and Web of Science. Two researchers will independently screen the titles, abstracts, and text of the returned articles for eligibility. Data charting and analysis will consist of a narrative description of the included studies, quantitative and qualitative findings relative to the aims of this scoping review. Gaps in the literature will be highlighted to inform future research, clinical practice, and policy.

Ethics and Dissemination: Ethical approval is not required as all data are available from public sources. The results of this scoping review will be disseminated through peer-reviewed publication, conference presentation, and social media.

Strengths and limitations of this study

- This scoping review will be the first synthesis of published and unpublished literature on intensive community and home treatments for eating disorders.
- This study will be conducted according to standardised guidelines for scoping reviews.

For peer review only - http://bmjopen.bmj.com/site/about/guidelines.xhtml

- The use of broad evidence sources and methodologies will increase the likelihood of the identification of all relevant studies.
- Screening and charting will be conducted by two independent researchers to ensure reliability.
- The studies screened and included in this review will be limited to publications in the English language.

For peer review only

For peer review only - http://bmjopen.bmj.com/site/about/guidelines.xhtml

INTRODUCTION

Eating disorders (ED) are serious and complex conditions associated with significant psychological, social, and economic burdens to the person, their carers, and society (1–3). Psychological comorbidities are the norm, prominently including depression and suicidality (e.g., 4,5). Likewise, medical complications, secondary to starvation and /or other ED symptoms (e.g. purging) are common (e.g., 7–9). Unsurprisingly, therefore, mortality rates for EDs, either due to physical health problems or due to suicide, are elevated relative to the general population and other psychiatric illnesses (1,9). The highest rates of mortality are observed for anorexia nervosa (AN), where risk is more than five times greater when compared to age- and gender-matched general populations (2,10). Accordingly, there is a need for treatment to begin promptly to minimise chronicity and improve the chances of a full recovery (1,11).

The demand for access to specialist services and intensive ED treatments seems to be increasing (12). Evidence indicates that while hospital admissions for other mental disorders have either stabilised or decreased worldwide, a different trend is apparent for EDs (13–15). For instance, in England hospital admissions for EDs doubled between 1998-2020 whilst admissions for other severe mental disorders decreased (16). This situation appears to have been further exacerbated by the ongoing Covid-19 pandemic. Internationally, research has indicated a rise in new ED presentations, increases in symptom severity and high levels of deterioration, relapse and comorbidities in those with established EDs (12,17,18). Unsurprisingly therefore, the need for ED treatment has increased, including the need for ED-related hospitalisations and other forms of intensive treatments for both children and adults (19–21).

Treatments for EDs are typically offered within a stepped-care model, where the intensity of treatment is matched to the severity of patient presentation. International guidelines recommend that the majority of ED patients should be offered specialist outpatient psychotherapy at the earliest opportunity. The recommended length of such outpatient psychotherapy is around 20 once-weekly sessions for bulimia nervosa (BN) or binge eating disorder (BED) and 20 to 40 sessions for those with AN (22). For those who do not adequately respond to outpatient care or whose physical health significantly deteriorates, specialised intensive treatments are recommended (22–24). Common intensive treatment modalities include inpatient, day patient (also known as partial hospitalisation), and residential treatments. These intensive treatment settings share goals and structures: treatment is multidisciplinary,

For peer review only - http://bmjopen.bmj.com/site/about/guidelines.xhtml

and patients in either treatment setting receive meal support, nutritional education, and individual and group psychotherapy (25). The key difference between the settings is that day patient treatment allows for patients to return home in the evening and on weekends.

Clinical guidelines for ED inpatient admission length and discharge criteria vary across EDs and countries. Decisions surrounding the length of hospital or inpatient stay for ED are generally complex, particularly for AN due to the associated psychiatric and medical complications. Data from specialised ED inpatient units in Scotland showed that the mean length of stay (LoS) was about 16 weeks for adults and 20 weeks for adolescents (26). A retrospective observational study using hospital admission data between 2000 and 2014 in Portugal showed that the median LoS was longest for AN, followed by unspecified ED and BN. Moreover, patients with AN had the highest readmission rates and hospitalisation costs (27). Recently, Kan and colleagues (28) conducted a comprehensive systematic review and meta-analysis on the LoS for AN. Their findings have demonstrated that the internationally pooled mean LoS in specialist inpatient services was about 11 weeks. However, the pooled mean LoS for AN appeared to be much longer in Europe (~15 weeks) than the United States (~7 weeks) and the other countries (~12 weeks). Furthermore, age and BMI were found to be significant moderators of the pooled mean LoS in both treatment settings, indicating that the higher the age and lower the BMI, the longer the stay. However, the overall LoS has decreased over time.

Day patient treatment is typically delivered 6-10 hours per day and 3 to 7 days per week depending on patients' needs and the resources of the services (29–31). The typical treatment duration mainly falls between 10 and 16 weeks across EDs (32); consistent with this, the international pooled mean LoS for AN is around 12 weeks (28). However, LoS can actually vary between 3 and 39 weeks across EDs. For instance, the mean day patient treatment LoS is longer for AN than ARFID, though the mean LoS is similar per diagnosis between inpatient and day patient services (28,33). Thus, it appears that day patient treatment programme structures vary widely between services depending on available resources, guiding theoretical models, treatment aims, and symptom severity (31,34).

Evidence demonstrates that inpatient treatment is associated with the highest costs of any ED treatment (25), consistently found to be more costly that both day patient (29,35,36) and specialist outpatient treatment (37). High inpatient treatment costs are especially relevant to

For peer review only - http://bmjopen.bmj.com/site/about/guidelines.xhtml

For peer review only

those with AN, as their often high medical risk and the time it takes to refeed those with very low BMIs requires a longer LoS (28,38,39). For instance, research conducted in Germany has demonstrated that over 50% of the yearly treatment costs for AN are due to hospitalisation (40). Though inpatient treatment costs are most pronounced in AN, the healthcare utilisation and treatment costs for BN and BED are also higher than those of the general population (41,42). Whilst day patient treatment is less costly than inpatient, a recent scoping review suggests that it is less cost-effective than outpatient treatment for those with EDs (43).

Although common intensive ED treatments, such as inpatient and day treatment, clearly have benefits for patients, including a focus on recovery, regular meal support, and improving physical health, they are also likely to bring additional challenges. For instance, inpatient treatment is associated with difficulties in maintaining relationships with friends and family, a sense of disconnection from real life (i.e. the potential for patients becoming institutionalised is high), and causes disruptions to education or work routines (44–46). In addition, the risk of relapse during the first months post-discharge is high (47–49). In contrast, day patient treatment allows patients to better maintain their relationship to the real world whilst receiving intensive treatment. However, just like inpatient treatment, attending a day patient programme and thus being at the hospital 3 to 7 days a week has the potential for disrupting education, employment and leisure activities (50). In addition, similar unintended iatrogenic effects as in inpatient treatment may be found in day programmes, in that strict schedules and routines around eating and predictable menus may produce an environment that fosters patients' inflexibility and reduces personal agency and self-efficacy (43). Moreover, outside urban areas with good transport connections, the daily commute to a day patient service can be challenging or impossible (e.g., 53). For example, the Mental Welfare Commission has reported that in Scotland, day patient services are geographically inequitable, leading to a particular difficulty in accessing support in the community for those living in rural areas (52). Lastly, day patient treatment can also place significant burdens on patients' families, e.g. through disruptions to their daily routines and them having to carry increased responsibility for acutely unwell patients' physical and mental health (53,54). All in all, traditional institutionally based intensive treatments such as inpatient and day treatment are costly, have limited availability and are associated with a number of disadvantages for patients. Therefore, alternative and more flexible intensive treatment options need to be explored to improve patient and carer outcomes and treatment experiences, manage increased demand for intensive treatments and optimally employ scarce resources.

For peer review only - http://bmjopen.bmj.com/site/about/guidelines.xhtml

Intensive community and home-based treatments as alternatives to inpatient and day patient treatments have previously been explored for other severe mental disorders, such as psychosis and mood disorders, in response to the significant challenges associated with traditional institutional care (e.g. high treatment costs, long waiting periods, disruptions to daily life, institutionalisation). These treatments were designed to increase direct and easy access to care within an everyday life context (e.g., home, community centres and clinics) and minimise hospital (re-)admissions and days spent in inpatient units (55–57). Intensive community and home-based treatments are usually carried out by a multidisciplinary team and include several clinical contacts per week with flexibility in their provision, according to clinical need. Investigations of intensive community and home-based treatments have provided evidence for their effectiveness and usefulness for managing mental disorders that would normally be treated within an institutional setting. For instance, a naturalistic study on patients with acute mental disorders found that intensive home-based treatment is an effective and cost-effective alternative to inpatient treatment for reducing acute psychotic and depressive symptoms as well as clinical and functional impairment (58). Similarly, in a mixed psychiatric population intensive home-based treatment improved psychiatric symptoms, suicidality, general functioning, and caregiver burden (59). A randomised controlled trial of adolescents with severe mental health problems admitted to inpatient services, compared inpatient treatment as usual to early discharge followed by intensive community care. Overall outcomes were similar in both groups, but patients allocated to intensive community care were significantly less likely to report multiple episodes of self-harm (60). Furthermore, these care models have been shown to be a valuable alternative to inpatient treatment through avoiding hospital admissions (61), thereby decreasing hospital days and readmission rates (60,62). Overall, the evidence suggests that these treatment modalities are cost-effective, and have been to linked to positive clinical and social outcomes as well as rates of patient and carer satisfaction comparable to institutionally based intensive treatments (63–66). Additionally, the community-based nature of these treatment modalities may improve accessibility and circumvent some of the barriers to care outlined previously.

The possible advantages of intensive community and home-based approaches have been proposed for the treatment of EDs (e.g., 69–72), however no attempt has yet been made to systematically review this area of research. We therefore aim to map literature on intensive community and home treatments for EDs through focusing on their conceptualisation,

For peer review only - http://bmjopen.bmj.com/site/about/guidelines.xhtml

implementation and clinical outcomes. We further aim to identify knowledge gaps and provide directions for future research.

Research Questions

i. What is the extent of the available literature on intensive community and home-based treatments for EDs?
ii. How are intensive community- and home-based treatments for EDs conceptualised and implemented?
iii. What is the evidence on the efficacy, acceptability and cost-effectiveness of intensive community- and home-based treatments for EDs?

METHODS AND ANALYSIS

A scoping review will be conducted to map out the literature on intensive community and home-based treatments for ED. This study will follow the Preferred Reporting Items for Systematic Review and Meta-Analysis Protocol extension for Scoping Reviews checklist (PRISMA-ScR) (71) and the Joanna Briggs Institute (JBI) Reviewer's Manual (72).

Eligibility criteria

Population: This review will consider studies in patients with any type of ED diagnosis with any age and gender, including those with comorbid psychiatric diagnoses.

Concept: This review will consider any studies investigating intensive community and home-based treatments for EDs. We define intensive community treatment as any treatment offering more than two therapeutic contacts per week, excluding mere physical monitoring contacts. This intensity cut-off was used as some outpatient therapies for EDs initially offer 2 sessions per week.

Context: This review will consider studies conducted in any geographical context or any community or home setting.

Types of evidence source: This review will consider studies with all types of research designs (e.g., randomised controlled trials, clinically controlled studies, quasi-experimental and case

For peer review only - http://bmjopen.bmj.com/site/about/guidelines.xhtml

control studies, observational studies, case studies) and studies using quantitative, qualitative, or mixed methods. Ongoing clinical trials listed in trials registries will also be considered in order to explore further how intensive community and home treatments are being conceptualised and studied. Evidence from the grey literature will also be considered. Only studies published in English will be included.

Studies will be excluded if they (i) include mixed psychiatric populations, (ii) describe non-intensive treatments, and (iii) describe institution/hospital-based treatments (i.e., inpatient and day patient).

Search strategy

The literature search will be conducted on four main databases: PubMed, PsycInfo, MEDLINE, Web of Science. Sources of unpublished studies and of grey literature will include Scopus, Google Scholar, US ClinicalTrials.gov, WHO International clinical trials registry platform search portal, ISRCTN Registry, and the European Union Clinical Trials Register. Finally, we will conduct a reference list search on the final included studies to identify any additional relevant studies.

A limited initial search was conducted in PubMed by Bİ in February 2022, to identify relevant keywords and develop a search strategy. Then, a preliminary search strategy was developed by Bİ and MP and reviewed by US. The search strategy will be tailored to each database by Bİ and MP, and the search will be conducted on studies published until 1st June 2022. The search terms will include variations of "feeding and eating disorders", "community treatment", "intensive outpatient", and "home treatment". A pilot search conducted on MEDLINE on 14/04/2022 is presented in the Supplemental material.

Study selection

After completing the searches, references will be uploaded to Endnote citation management software, and duplicates will be removed. Titles and abstracts will be reviewed and remaining full-text citations will be screened for eligibility by Bİ and MP independently. In the case of any discrepancies, the reviewers will meet to discuss relevant studies. If the two reviewers cannot reach an agreement on the eligibility of a study, this disagreement will be resolved through consultation with US. Rayyan software will be used in this process.

For peer review only - http://bmjopen.bmj.com/site/about/guidelines.xhtml

Data charting

The data charting form developed by the present authors will be used to extract the key information relevant to study aims. The draft data charting form is presented in Table 1. This form might be updated during the final review process in order to ensure all relevant data are accurately captured.

The data will be charted by Bİ and MP independently for each of the studies, to assess if they meet inclusion criteria. If any disagreement arises, the reviewers will discuss to reach a consensus. US will be consulted in the case of uncertainty or unsolved disagreement.

Table 1 Draft Data Charting From

Data Item	Data
Author(s)	
Publication Year	
Location & Country	
Study Design	
Study Population and Sample Size	
Intervention Type	
Intervention Length & Dosage	
Intervention Delivery Mode	
Professional(s) Delivering the Intervention	
Admission & Discharge Criteria (if available)	
Main findings	

Data analysis and synthesis

The detailed information regarding the selection procedure will be presented in a PRISMA Flow Diagram. A summary of the studies using the data charting form will be provided in a table with an accompanying narrative description relating the research aims of this review. Where relevant, common and distinctive characteristics of the treatment modalities will be identified. The strength of the evidence will be examined using the JBI Levels of Evidence for Effectiveness: Level 1 (high) - experimental designs, Level 2 - quasi-experimental designs, Level 3 - observational – analytic designs, Level 4 - observational–descriptive and Level 5

For peer review only - http://bmjopen.bmj.com/site/about/guidelines.xhtml

(low) - Expert Opinion. To guide clinical applications, future research and policy makers, knowledge gaps will be highlighted.

Patient and public involvement

No patients or members of the public were involved in the development of this protocol or will be involved in the final scoping review. However, a recent report by BEAT (73), the UK's leading ED charity for patients and carers, entitled: "Intensive day- and home-based treatment for eating disorders: an effective and less expensive alternative to inpatient care" highlighted that patients and families see intensive community and home-based treatment modalities as more acceptable and empowering alternatives to traditional inpatient treatment. The report recommends that such community-based provisions are made more widely available and also highlights the need for research in this area.

ETHICS AND DISSEMINATION

Ethical approval will not be required for this review as the data will be obtained from publicly available sources. Dissemination of the review's findings will be performed through peer-reviewed publication and through conference presentations. Additionally, we will use social media (i.e., Twitter and Instagram) to reach the wider public.

Summary and Conclusion

To our knowledge, this will be the first review to map out the literature on non-institutional (i.e. community and home based) intensive treatments for ED. The findings will provide an overview on the extent, characteristics, clinical outcomes, and cost-effectiveness of these non-institutionally based intensive treatments. Additionally, this review will provide insights into patient and carer experiences with these interventions. We expect that this review will suggest future directions for designing new intensive treatment services or improving the existing ones, as well as for developing research priorities and policies.

Authors' contributions: US conceived of the idea for this scoping review. Bİ developed the research questions, search strategy, data charting tool and data analysis plan with support from MP. Bİ and MP drafted the manuscript. US supervised the protocol development and critically reviewed the manuscript. All authors have read and approved final version of the manuscript.

For peer review only - http://bmjopen.bmj.com/site/about/guidelines.xhtml

Funding: US receives salary support from the National Institute for Health Research (NIHR) Biomedical Research Centre for Mental Health, South London and Maudsley NHS Foundation Trust and Institute of Psychiatry, Psychology and Neuroscience, King's College London.

Competing interests: None declared.

Patient consent for publication: Not required.

For peer review only

For peer review only - http://bmjopen.bmj.com/site/about/guidelines.xhtml

REFERENCES

1. Schaumberg K, Welch E, Breithaupt L, Hübel C, Baker JH, Munn-Chernoff MA, et al. The Science Behind the Academy for Eating Disorders' Nine Truths About Eating Disorders. Eur Eat Disord Rev. 2017 Nov;25(6):432–50.
2. van Hoeken D, Hoek HW. Review of the burden of eating disorders: mortality, disability, costs, quality of life, and family burden. Curr Opin Psychiatry. 2020;33(6).
3. Treasure J, Duarte TA, Schmidt U. Eating disorders. Lancet (London, England). 2020 Mar;395(10227):899–911.
4. Keski-Rahkonen A, Mustelin L. Epidemiology of eating disorders in Europe: prevalence, incidence, comorbidity, course, consequences, and risk factors. Curr Opin Psychiatry. 2016/09/24. 2016;29(6):340–5.
5. Portzky G, van Heeringen K, Vervaet M. Attempted suicide in patients with eating disorders. Vol. 35, Crisis: The Journal of Crisis Intervention and Suicide Prevention. Portzky, Gwendolyn: Unit for Suicide Research–Ghent University, University Hospital Ghent, 1K12F, De Pintelaan 185, Ghent, Belgium, 9000, gwendolyn.portzky@ugent.be: Hogrefe Publishing; 2014. p. 378–87.
6. Giel KE, Bulik CM, Fernandez-Aranda F, Hay P, Keski-Rahkonen A, Schag K, et al. Binge eating disorder. Nat Rev Dis Prim. 2022;8(1):16.
7. Mehler PS, Rylander M. Bulimia Nervosa - medical complications. J Eat Disord. 2015/04/14. 2015;3:12.
8. Treasure J, Zipfel S, Micali N, Wade T, Stice E, Claudino A, et al. Anorexia nervosa. Nat Rev Dis Prim. 2015;1(1):15075.
9. Fichter MM, Quadflieg N. Mortality in eating disorders - results of a large prospective clinical longitudinal study. Int J Eat Disord. 2016 Apr;49(4):391–401.
10. Arcelus J, Mitchell AJ, Wales J, Nielsen S. Mortality Rates in Patients With Anorexia Nervosa and Other Eating Disorders: A Meta-analysis of 36 Studies. Arch Gen Psychiatry. 2011 Jul;68(7):724–31.
11. Wonderlich S, Mitchell JE, Crosby RD, Myers TC, Kadlec K, LaHaise K, et al. Minimizing and treating chronicity in the eating disorders: A clinical overview. Int J Eat Disord. 2012 May;45(4):467–75.
12. Linardon J, Messer M, Rodgers RF, Fuller-Tyszkiewicz M. A systematic scoping review of research on COVID-19 impacts on eating disorders: A critical appraisal of the evidence and recommendations for the field. Int J Eat Disord. 2022 Jan;55(1):3–38.
13. de Graaf R, ten Have M, van Gool C, van Dorsselaer S. Prevalence of mental disorders

For peer review only - http://bmjopen.bmj.com/site/about/guidelines.xhtml

and trends from 1996 to 2009. Results from the Netherlands Mental Health Survey and Incidence Study-2 . Soc Psychiatry Psychiatr Epidemiol. 2012;47(2):203–13.

14. Baxter AJ, Scott KM, Ferrari AJ, Norman RE, Vos T, Whiteford HA. Challenging the myth of an "epidemic" of common mental disorders: Trends in the global prevalence of anxiety and depression between 1990 and 2010. Depress Anxiety. 2014 Jun;31(6):506–16.

15. Green BH, Griffiths EC. Hospital admission and community treatment of mental disorders in England from 1998 to 2012. Gen Hosp Psychiatry. 2014;36(4):442–8.

16. Degli Esposti M, Ziauddeen H, Bowes L, Reeves A, Chekroud AM, Humphreys DK, et al. Trends in inpatient care for psychiatric disorders in NHS hospitals across England, 1998/99–2019/20: an observational time series analysis. Soc Psychiatry Psychiatr Epidemiol. 2021;

17. Schlegl S, Maier J, Meule A, Voderholzer U. Eating disorders in times of the COVID-19 pandemic—Results from an online survey of patients with anorexia nervosa. Int J Eat Disord. 2020 Nov;53(11):1791–800.

18. Giel KE, Schurr M, Zipfel S, Junne F, Schag K. Eating behaviour and symptom trajectories in patients with a history of binge eating disorder during COVID-19 pandemic. Eur Eat Disord Rev. 2021 Jul;29(4):657–62.

19. Asch DA, Buresh J, Allison KC, Islam N, Sheils NE, Doshi JA, et al. Trends in US Patients Receiving Care for Eating Disorders and Other Common Behavioral Health Conditions Before and During the COVID-19 Pandemic. JAMA Netw Open. 2021 Nov;4(11):e2134913–e2134913.

20. Lin JA, Hartman-Munick SM, Kells MR, Milliren CE, Slater WA, Woods ER, et al. The Impact of the COVID-19 Pandemic on the Number of Adolescents/Young Adults Seeking Eating Disorder-Related Care. J Adolesc Heal. 2021;69(4):660–3.

21. Toulany A, Kurdyak P, Guttmann A, Stukel TA, Fu L, Strauss R, et al. Acute Care Visits for Eating Disorders Among Children and Adolescents After the Onset of the COVID-19 Pandemic. J Adolesc Heal. 2022;70(1):42–7.

22. NICE. Eating disorders: Recognition and treatment. NICE guideline. London: National Institute for Health and Care Excellence; 2017.

23. American Psychiatric Association. Treatment of patients with eating disorders,third edition. Am J Psychiatry. 2006 Jul;163(7 Suppl):4–54.

24. Heruc G, Hurst K, Casey A, Fleming K, Freeman J, Fursland A, et al. ANZAED eating disorder treatment principles and general clinical practice and training standards. J Eat

For peer review only - http://bmjopen.bmj.com/site/about/guidelines.xhtml

Disord. 2020;8(1):63.
25. Madden S, Hay P, Touyz S. Systematic review of evidence for different treatment settings in anorexia nervosa. World J psychiatry. 2015 Mar;5(1):147–53.
26. Morris J, Simpson A V, Voy SJ. Length of stay of inpatients with eating disorders. Vol. 22, Clinical Psychology & Psychotherapy. Morris, Jane: Eden Unit, Royal Cornhill Hospital, Aberdeen, Scotland, AB25 2ZH, janemorris1@nhs.net: John Wiley & Sons; 2015. p. 45–53.
27. Cruz AM, Gonçalves-Pinho M, Santos JV, Coutinho F, Brandão I, Freitas A. Eating disorders—Related hospitalizations in Portugal: A nationwide study from 2000 to 2014. Int J Eat Disord. 2018 Oct;51(10):1201–6.
28. Kan C, Hawkings Y-R, Cribben H, Treasure J. Length of stay for anorexia nervosa: Systematic review and meta-analysis. Eur Eat Disord Rev. 2021 May;29(3):371–92.
29. Zipfel S, Reas DL, Thornton C, Olmsted MP, Williamson DA, Gerlinghoff M, et al. Day hospitalization programs for eating disorders: A systematic review of the literature. Int J Eat Disord. 2002 Mar;31(2):105–17.
30. Brown TA, Cusack A, Anderson LK, Trim J, Nakamura T, Trunko ME, et al. Efficacy of a partial hospital programme for adults with eating disorders. Eur Eat Disord Rev. 2018 May;26(3):241–52.
31. Abbate-Daga G, Gramaglia C, Preda S, Comba E, Brustolin A, Fassino S. Day hospital programmes for eating disorders: A review of the similarities, differences and goals. Eat Weight Disord - Stud Anorexia, Bulim Obes. 2009;14(2):e31–41.
32. Friedman K, Ramirez AL, Murray SB, Anderson LK, Cusack A, Boutelle KN, et al. A Narrative Review of Outcome Studies for Residential and Partial Hospital-based Treatment of Eating Disorders. Eur Eat Disord Rev. 2016 Jul;24(4):263–76.
33. Makhzoumi SH, Schreyer CC, Hansen JL, Laddaran LA, Redgrave GW, Guarda AS. Hospital course of underweight youth with ARFID treated with a meal-based behavioral protocol in an inpatient-partial hospitalization program for eating disorders. Int J Eat Disord. 2019 Apr;52(4):428–34.
34. Baudinet J, Simic M. Adolescent Eating Disorder Day Programme Treatment Models and Outcomes: A Systematic Scoping Review. Front psychiatry. 2021;12:652604.
35. Guarda AS, Schreyer CC, Fischer LK, Hansen JL, Coughlin JW, Kaminsky MJ, et al. Intensive treatment for adults with anorexia nervosa: The cost of weight restoration. Int J Eat Disord. 2017 Mar;50(3):302–6.
36. Williamson DA, Thaw JM, Varnado-Sullivan PJ. Cost-effectiveness analysis of a

For peer review only - http://bmjopen.bmj.com/site/about/guidelines.xhtml

hospital-based cognitive-behavioral treatment program for eating disorders. Behav Ther. 2001;32(3):459–77.

37. Byford S, Barrett B, Roberts C, Clark A, Edwards V, Smethurst N, et al. Economic evaluation of a randomised controlled trial for anorexia nervosa in adolescents. Br J Psychiatry. 2018/01/02. 2007;191(5):436–40.

38. Haas L, Stargardt T, Schreyoegg J, Schlösser R, Danzer G, Klapp BF. Inpatient costs and predictors of costs in the psychosomatic treatment of anorexia nervosa. Int J Eat Disord. 2012 Mar;45(2):214–21.

39. Toulany A, Wong M, Katzman DK, Akseer N, Steinegger C, Hancock-Howard RL, et al. Cost analysis of inpatient treatment of anorexia nervosa in adolescents: hospital and caregiver perspectives. C Open. 2015 Apr;3(2):E192 LP-E197.

40. Stuhldreher N, Wild B, König H-H, Konnopka A, Zipfel S, Herzog W. Determinants of direct and indirect costs in anorexia nervosa. Int J Eat Disord. 2015 Jan;48(1):139–46.

41. Ágh T, Kovács G, Supina D, Pawaskar M, Herman BK, Vokó Z, et al. A systematic review of the health-related quality of life and economic burdens of anorexia nervosa, bulimia nervosa, and binge eating disorder. Eat Weight Disord - Stud Anorexia, Bulim Obes. 2016;21(3):353–64.

42. Samnaliev M, Noh HL, Sonneville KR, Austin SB. The economic burden of eating disorders and related mental health comorbidities: An exploratory analysis using the U.S. Medical Expenditures Panel Survey. Prev Med Reports. 2015;2:32–4.

43. Ali SI, Bodnar E, Gamberg S, Bartel SJ, Waller G, Nunes A, et al. The costs and benefits of intensive day treatment programs and outpatient treatments for eating disorders: An idea worth researching. Int J Eat Disord. 2021 Jul;54(7):1099–105.

44. Offord A, Turner H, Cooper M. Adolescent inpatient treatment for anorexia nervosa: a qualitative study exploring young adults' retrospective views of treatment and discharge. Eur Eat Disord Rev. 2006 Nov;14(6):377–87.

45. Bezance J, Holliday J. Adolescents with Anorexia Nervosa Have Their Say: A Review of Qualitative Studies on Treatment and Recovery from Anorexia Nervosa. Eur Eat Disord Rev. 2013 Sep;21(5):352–60.

46. Elran-Barak R, Barak A, Cohen-Ashkenazi S, Schifter T. Barriers to hospital admission among people with eating disorders: A qualitative internet-based study. Int J Ment Health Nurs. 2018 Dec;27(6):1784–92.

47. Richard M, Bauer S, Kordy H. Relapse in anorexia and bulimia nervosa—a 2.5-year

For peer review only - http://bmjopen.bmj.com/site/about/guidelines.xhtml

For peer review only

follow-up study. Eur Eat Disord Rev. 2005 May;13(3):180–90.

48. Walsh BT, Xu T, Wang Y, Attia E, Kaplan AS. Time Course of Relapse Following Acute Treatment for Anorexia Nervosa. Am J Psychiatry. 2021 Jun;178(9):848–53.

49. Khalsa SS, Portnoff LC, McCurdy-McKinnon D, Feusner JD. What happens after treatment? A systematic review of relapse, remission, and recovery in anorexia nervosa. J Eat Disord. 2017;5(1):20.

50. Matthews K, Gordon L, van Beusekom J, Sheffield J, Painter E, Bellair R, et al. A day treatment program for adults with eating disorders: staff and patient experiences in implementation. J Eat Disord. 2019;7(1):21.

51. Nikendei C, Haitz M, Huber J, Ehrenthal JC, Herzog W, Schauenburg H, et al. Day clinic and inpatient psychotherapy of depression (DIP-D): qualitative results from a randomized controlled study. Int J Ment Health Syst. 2016;10(1):41.

52. Mental Welfare Commission. Mental Welfare Commission for Scotland. Mapping of Eating Disorder Services across Scotland. 2020.

53. Fox JR, Dean M, Whittlesea A. The Experience of Caring For or Living with an Individual with an Eating Disorder: A Meta-Synthesis of Qualitative Studies. Clin Psychol Psychother. 2017 Jan;24(1):103–25.

54. Robinson I, Stoyel H, Robinson P. "If she had broken her leg she would not have waited in agony for 9 months": Caregiver's experiences of eating disorder treatment. Eur Eat Disord Rev. 2020 Nov;28(6):750–65.

55. Burns T. Community mental health teams. Psychiatry. 2004;3(9):11–4.

56. Johnson S. Crisis resolution and intensive home treatment teams. Psychiatry. 2004;3(9):22–5.

57. Johnson S. Crisis resolution and home treatment teams: an evolving model. Adv Psychiatr Treat. 2018/01/02. 2013;19(2):115–23.

58. Kilian R, Becker T, Frasch K. Effectiveness and cost-effectiveness of home treatment compared with inpatient care for patients with acute mental disorders in a rural catchment area in Germany. Neurol Psychiatry Brain Res. 2016;22(2):81–6.

59. Gotink RA, Bergsma G, Hoogwegt MT, Mulder CL, Van Gool AR. Intensive home treatment: een eerste observationele evaluatie. Tijdschr Psychiatr. 2017;59(11):713–9.

60. Ougrin D, Corrigall R, Stahl D, Poole J, Zundel T, Wait M, et al. Supported discharge service versus inpatient care evaluation (SITE): a randomised controlled trial comparing effectiveness of an intensive community care service versus inpatient treatment as usual for adolescents with severe psychiatric disorders: self-h. Eur Child

For peer review only - http://bmjopen.bmj.com/site/about/guidelines.xhtml

For peer review only

Adolesc Psychiatry. 2021;30(9):1427–36.

61. Huang HCH, Taylor M, Carmichael A. The outcomes of home treatment for schizophrenia. BJPsych Bull. 2018/08/06. 2018 Dec;42(6):238–42.

62. Mötteli S, Schori D, Schmidt H, Seifritz E, Jäger M. Utilization and Effectiveness of Home Treatment for People With Acute Severe Mental Illness: A Propensity-Score Matching Analysis of 19 Months of Observation . Vol. 9, Frontiers in Psychiatry . 2018.

63. Boege I, Corpus N, Schepker R, Kilian R, Fegert JM. Cost-effectiveness of intensive home treatment enhanced by inpatient treatment elements in child and adolescent psychiatry in Germany: A randomised trial. Eur Psychiatry. 2020/04/15. 2015;30(5):583–9.

64. Stulz N, Wyder L, Maeck L, Hilpert M, Lerzer H, Zander E, et al. Home treatment for acute mental healthcare: randomised controlled trial. Br J Psychiatry. 2019/03/13. 2020;216(6):323–30.

65. Graf D, Lerch S, Böhnke U, Reichl C, Kindler J, Koenig J, et al. Treatment outcome of an intensive psychiatric home treatment for children and adolescents: a non-randomized controlled pilot evaluation. Eur Child Adolesc Psychiatry. 2021;

66. Kwok KHR, Yuan SNV, Ougrin D. Review: Alternatives to inpatient care for children and adolescents with mental health disorders. Child Adolesc Ment Health. 2016 Feb;21(1):3–10.

67. Herpertz-Dahlmann B. Intensive Treatments in Adolescent Anorexia Nervosa. Vol. 13, Nutrients . 2021.

68. Holmes B, Frampton I, Clark-Stone S. Evaluation of the outcomes following home treatment for anorexia nervosa [Unpublished audit report]. 2018.

69. Munro C, Thomson V, Corr J, Randell L, Davies JE, Gittoes C, et al. A new service model for the treatment of severe anorexia nervosa in the community: the Anorexia Nervosa Intensive Treatment Team. Psychiatr Bull. 2018/01/02. 2014;38(5):220–5.

70. Pauli D, Flütsch N, Hilti N, Schräer C, Soumana M, Häberling I, et al. Home treatment as an add-on to family-based treatment in adolescents with anorexia nervosa: A pilot study. Eur Eat Disord Rev [Internet]. 2022 Mar 1;30(2):168–77. Available from: https://doi.org/10.1002/erv.2882

71. Tricco AC, Lillie E, Zarin W, O’Brien KK, Colquhoun H, Levac D, et al. PRISMA Extension for Scoping Reviews (PRISMA-ScR): Checklist and Explanation. Ann Intern Med. 2018 Sep;169(7):467–73.

For peer review only - http://bmjopen.bmj.com/site/about/guidelines.xhtml

72. Peters MDJ, Godfrey C, McInerney P, Munn Z, Tricco AC, Khalil H. JBI Manual for Evidence Synthesis, JBI, 2020. In: Aromataris E, Munn Z, editors. 2020.

73. Beat. Intensive day- and home-based treatment for eating disorders: an effective and less expensive alternative to inpatient care. 2019.

For peer review only

For peer review only - http://bmjopen.bmj.com/site/about/guidelines.xhtml

Supplemental material

Pilot search on Ovid MEDLINE

	Query	Results
**#1**	exp Community Mental Health Centers/ or exp Community Mental Health Services/	21905
**#2**	("community care*" or "community treat*" or "intensive community" or "intensive outpatient*" or "home care*" or "home treat*" or "home feeding*").mp.	63306
**#3**	#1 OR #2	82885
**#4**	exp "Feeding and Eating Disorders"/	34241
**#5**	(eating adj3 disorder*).mp.	32467
**#6**	anorex*.mp.	40508
**#7**	bulimi*.mp.	11911
**#8**	"binge eating*".mp.	6902
**#9**	("Avoidant Restrictive Food Intake Disorder" or ARFID).mp.	363
**#10**	#4 OR #5 OR #6 OR #7 OR #8 OR #9	68413
**#11**	#3 AND #10	224

For peer review only - http://bmjopen.bmj.com/site/about/guidelines.xhtml

Preferred Reporting Items for Systematic reviews and Meta-Analyses extension for Scoping Reviews (PRISMA-ScR) Checklist

SECTION	ITEM	PRISMA-ScR CHECKLIST ITEM	REPORTED ON PAGE #
**TITLE**			
Title	1	Identify the report as a scoping review.	1
**ABSTRACT**			
Structured summary	2	Provide a structured summary that includes (as applicable): background, objectives, eligibility criteria, sources of evidence, charting methods, results, and conclusions that relate to the review questions and objectives.	2
**INTRODUCTION**			
Rationale	3	Describe the rationale for the review in the context of what is already known. Explain why the review questions/objectives lend themselves to a scoping review approach.	4 to 8
Objectives	4	Provide an explicit statement of the questions and objectives being addressed with reference to their key elements (e.g., population or participants, concepts, and context) or other relevant key elements used to conceptualize the review questions and/or objectives.	8
**METHODS**			
Protocol and registration	5	Indicate whether a review protocol exists; state if and where it can be accessed (e.g., a Web address); and if available, provide registration information, including the registration number.	N/A
Eligibility criteria	6	Specify characteristics of the sources of evidence used as eligibility criteria (e.g., years considered, language, and publication status), and provide a rationale.	8 & 9
Information sources*	7	Describe all information sources in the search (e.g., databases with dates of coverage and contact with authors to identify additional sources), as well as the date the most recent search was executed.	9
Search	8	Present the full electronic search strategy for at least 1 database, including any limits used, such that it could be repeated.	Supplement
Selection of sources of evidence†	9	State the process for selecting sources of evidence (i.e., screening and eligibility) included in the scoping review.	9
Data charting process‡	10	Describe the methods of charting data from the included sources of evidence (e.g., calibrated forms or forms that have been tested by the team before their use, and whether data charting was done independently or in duplicate) and any processes for obtaining and confirming data from investigators.	10
Data items	11	List and define all variables for which data were sought and any assumptions and simplifications made.	N/A
Critical appraisal of individual sources of evidence§	12	If done, provide a rationale for conducting a critical appraisal of included sources of evidence; describe the methods used and how this information was used in any data synthesis (if appropriate).	N/A

St. Michael's
Inspired Care.
Inspiring Science.

For peer review only - http://bmjopen.bmj.com/site/about/guidelines.xhtml

SECTION	ITEM	PRISMA-ScR CHECKLIST ITEM	REPORTED ON PAGE #
Synthesis of results	13	Describe the methods of handling and summarizing the data that were charted.	10&11
**RESULTS**			
Selection of sources of evidence	14	Give numbers of sources of evidence screened, assessed for eligibility, and included in the review, with reasons for exclusions at each stage, ideally using a flow diagram.	N/A
Characteristics of sources of evidence	15	For each source of evidence, present characteristics for which data were charted and provide the citations.	N/A
Critical appraisal within sources of evidence	16	If done, present data on critical appraisal of included sources of evidence (see item 12).	N/A
Results of individual sources of evidence	17	For each included source of evidence, present the relevant data that were charted that relate to the review questions and objectives.	N/A
Synthesis of results	18	Summarize and/or present the charting results as they relate to the review questions and objectives.	N/A
**DISCUSSION**			
Summary of evidence	19	Summarize the main results (including an overview of concepts, themes, and types of evidence available), link to the review questions and objectives, and consider the relevance to key groups.	N/A
Limitations	20	Discuss the limitations of the scoping review process.	N/A
Conclusions	21	Provide a general interpretation of the results with respect to the review questions and objectives, as well as potential implications and/or next steps.	N/A
**FUNDING**			
Funding	22	Describe sources of funding for the included sources of evidence, as well as sources of funding for the scoping review. Describe the role of the funders of the scoping review.	12

JBI = Joanna Briggs Institute; PRISMA-ScR = Preferred Reporting Items for Systematic reviews and Meta-Analyses extension for Scoping Reviews.
* Where *sources of evidence* (see second footnote) are compiled from, such as bibliographic databases, social media platforms, and Web sites.
† A more inclusive/heterogeneous term used to account for the different types of evidence or data sources (e.g., quantitative and/or qualitative research, expert opinion, and policy documents) that may be eligible in a scoping review as opposed to only studies. This is not to be confused with *information sources* (see first footnote).
‡ The frameworks by Arksey and O'Malley (6) and Levac and colleagues (7) and the JBI guidance (4, 5) refer to the process of data extraction in a scoping review as data charting.
§ The process of systematically examining research evidence to assess its validity, results, and relevance before using it to inform a decision. This term is used for items 12 and 19 instead of "risk of bias" (which is more applicable to systematic reviews of interventions) to include and acknowledge the various sources of evidence that may be used in a scoping review (e.g., quantitative and/or qualitative research, expert opinion, and policy document).

From: Tricco AC, Lillie E, Zarin W, O'Brien KK, Colquhoun H, Levac D, et al. PRISMA Extension for Scoping Reviews (PRISMAScR): Checklist and Explanation. Ann Intern Med. 2018;169:467–473. doi: 10.7326/M18-0850.

For peer review only - http://bmjopen.bmj.com/site/about/guidelines.xhtml

BMJ Open

Intensive Community and Home-Based Treatments for Eating Disorders: A Scoping Review Study Protocol

Journal:	*BMJ Open*
Manuscript ID	bmjopen-2022-064243.R1
Article Type:	Protocol
Date Submitted by the Author:	24-Dec-2022

Complete List of Authors:	İnce, Başak; King's College London Institute of Psychiatry Psychology and Neuroscience, Department of Psychological Medicine - Section of Eating Disorders<br>Phillips, Matthew; King's College London Institute of Psychiatry Psychology and Neuroscience, Department of Psychological Medicine - Section of Eating Disorders<br>Schmidt, Ulrike; King's College London Institute of Psychiatry Psychology and Neuroscience, Department of Psychological Medicine - Section of Eating Disorders; South London and Maudsley NHS Foundation Trust, Eating Disorder Outpatients Service

**Primary Subject Heading**:	Mental health
Secondary Subject Heading:	Evidence based practice, Health services research
Keywords:	Eating disorders < PSYCHIATRY, Adult psychiatry < PSYCHIATRY, Child & adolescent psychiatry < PSYCHIATRY, PUBLIC HEALTH

SCHOLARONE™
Manuscripts

Intensive Community and Home-Based Treatments for Eating Disorders: A Scoping Review Study Protocol

Başak İnce [1], Matthew Phillips [1], Ulrike Schmidt [1,2]

[1] Department of Psychological Medicine, Section of Eating Disorders, Institute of Psychiatry, Psychology & Neuroscience, King's College London, London, UK

[2] Eating Disorder Outpatients Service, South London and Maudsley NHS Foundation Trust, London, UK

Corresponding author: Başak İnce (basak.ince@kcl.ac.uk)

Department of Psychological Medicine, Section of Eating Disorders, Institute of Psychiatry, Psychology & Neuroscience, King's College London, London SE5 8AF, UK.

ORCID:

Başak İnce: http://orcid.org/0000-0003-1177-3490

Matthew Phillips: https://orcid.org/0000-0001-8250-5560

Ulrike Schmidt: http://orcid.org/0000-0003-1335-1937

Word count: 2920

For peer review only

ABSTRACT

Introduction: Institutionally based intensive treatment modalities (inpatient, day patient, and residential treatments) for eating disorders (ED) are associated with high treatment costs and significant challenges for patients and carers, including access difficulties and disruption to daily routines. Intensive community and home-based treatments have been suggested as alternatives to institutionally based intensive treatments for other severe mental illnesses, with promising clinical, social, and health economic outcomes. The possible advantages of these treatments have been proposed for EDs, but this emerging area of research has not yet been systematically investigated. This scoping review aims to map the available literature on intensive community and home treatments for EDs, focusing on their conceptualisation, implementation, and clinical outcomes.

Methods: This proposed scoping review will follow the Preferred Reporting Items for Systematic Review and Meta-Analysis Protocol extension for Scoping Reviews checklist and the Joanna Briggs Institute Reviewer's Manual. This review will include any peer-reviewed study concerning intensive community and home-based treatments for any EDs, with no restrictions on geographical context or study design. Grey literature will also be considered. The literature search will be conducted in four databases: PubMed, PsycInfo, MEDLINE, and Web of Science. Two researchers will independently screen the titles, abstracts, and text of the returned articles for eligibility. Data charting and analysis will consist of a narrative description of the included studies, quantitative and qualitative findings relative to the aims of this scoping review. Gaps in the literature will be highlighted to inform future research, clinical practice, and policy.

Ethics and Dissemination: Ethical approval is not required as all data are available from public sources. The results of this scoping review will be disseminated through peer-reviewed publication, conference presentation, and social media.

Strengths and limitations of this study

- This scoping review will be the first synthesis of published and unpublished literature on intensive community and home treatments for eating disorders.
- This study will be conducted according to standardised guidelines for scoping reviews.

For peer review only

For peer review only - http://bmjopen.bmj.com/site/about/guidelines.xhtml

- The use of broad evidence sources and methodologies will increase the likelihood of the identification of all relevant studies.
- Screening and charting will be conducted by two independent researchers to ensure reliability.
- The studies screened and included in this review will be limited to publications in the English language.

For peer review only

For peer review only - http://bmjopen.bmj.com/site/about/guidelines.xhtml

INTRODUCTION

Eating disorders (ED) are serious and complex conditions associated with significant psychological, social, and economic burdens to the person, their carers, and society (1–3). Psychological comorbidities are the norm, prominently including depression and suicidality (e.g., 4,5). Likewise, medical complications, secondary to starvation and /or other ED symptoms (e.g. purging) are common (e.g., 6–8). Unsurprisingly, therefore, mortality rates for EDs, either due to physical health problems or due to suicide, are elevated relative to the general population and other psychiatric illnesses (1,9). The highest rates of mortality are observed for anorexia nervosa (AN), where risk is more than five times greater when compared to age- and gender-matched general populations (2,10). Accordingly, there is a need for treatment to begin promptly to minimise chronicity and improve the chances of a full recovery (1,11).

The demand for access to specialist services and intensive ED treatments seems to be increasing (12). Evidence indicates that while hospital admissions for other mental disorders have either stabilised or decreased worldwide, a different trend is apparent for EDs (13–15). For instance, in England hospital admissions for EDs doubled between 1998-2020 whilst admissions for other severe mental disorders decreased (16). This situation appears to have been further exacerbated by the ongoing Covid-19 pandemic. Internationally, research has indicated a rise in new ED presentations, increases in symptom severity and high levels of deterioration, relapse and comorbidities in those with established EDs (12,17,18). Unsurprisingly therefore, the need for ED treatment has increased, including the need for ED-related hospitalisations and other forms of intensive treatments for both children and adults (19–21).

Treatments for EDs are typically offered within a stepped-care model, where the intensity of treatment is matched to the severity of patient presentation. International guidelines recommend that the majority of ED patients should be offered specialist outpatient psychotherapy at the earliest opportunity. The recommended length of such outpatient psychotherapy is around 20 once-weekly sessions for bulimia nervosa (BN) or binge eating disorder (BED) and 20 to 40 sessions for those with AN (22). For those who do not adequately respond to outpatient care or whose physical health significantly deteriorates, specialised intensive treatments are recommended (22–24). Common intensive treatment modalities include inpatient, day patient (also known as partial hospitalisation), and residential treatments. These intensive treatment settings share goals and structures: treatment is multidisciplinary,

For peer review only - http://bmjopen.bmj.com/site/about/guidelines.xhtml

and patients in either treatment setting receive meal support, nutritional education, and individual and group psychotherapy (25). The key difference between the settings is that day patient treatment allows for patients to return home in the evening and on weekends.

Clinical guidelines for ED inpatient admission length and discharge criteria vary across EDs and countries. Decisions surrounding the length of hospital or inpatient stay for ED are generally complex, particularly for AN due to the associated psychiatric and medical complications. Data from specialised ED inpatient units in Scotland showed that the mean length of stay (LoS) was about 16 weeks for adults and 20 weeks for adolescents (26). A retrospective observational study using hospital admission data between 2000 and 2014 in Portugal showed that the median LoS was longest for AN, followed by unspecified ED and BN. Moreover, patients with AN had the highest readmission rates and hospitalisation costs (27). Recently, Kan and colleagues (28) conducted a comprehensive systematic review and meta-analysis on the LoS for AN. Their findings have demonstrated that the internationally pooled mean LoS in specialist inpatient services was about 11 weeks. However, the pooled mean LoS for AN appeared to be much longer in Europe (~15 weeks) than the United States (~7 weeks) and the other countries (~12 weeks), which might be a result of differences between healthcare systems across countries. Furthermore, age and BMI were found to be significant moderators of the pooled mean LoS in both treatment settings, indicating that the higher the age and lower the BMI, the longer the stay. However, the overall LoS has decreased over time.

Day patient treatment is typically delivered 6-10 hours per day and 3 to 7 days per week depending on patients' needs and the resources of the services (29–31). The typical treatment duration mainly falls between 10 and 16 weeks across EDs (32); consistent with this, the international pooled mean LoS for AN is around 12 weeks (28). However, LoS can actually vary between 3 and 39 weeks across EDs. For instance, the mean day patient treatment LoS is longer for AN than avoidant/restrictive food intake disorder (ARFID), though the mean LoS is similar per diagnosis between inpatient and day patient services (28,33). Thus, it appears that day patient treatment programme structures vary widely between services depending on available resources, guiding theoretical models, treatment aims, and symptom severity (31,34).

Evidence demonstrates that inpatient treatment is associated with the highest costs of any ED treatment (25), consistently found to be more costly than both day patient (29,35,36) and specialist outpatient treatment (37). High inpatient treatment costs are especially relevant to

For peer review only - http://bmjopen.bmj.com/site/about/guidelines.xhtml

those with AN, as their often high medical risk and the time it takes to refeed those with very low BMIs requires a longer LoS (28,38,39). For instance, research conducted in Germany has demonstrated that over 50% of the yearly treatment costs for AN are due to hospitalisation (40). Though inpatient treatment costs are most pronounced in AN, the healthcare utilisation and treatment costs for BN and BED are also higher than those of the general population (41,42). For example, a recent paper (43) reported a near-fourfold increase in mean healthcare spending among patients with eating disorders ($29,456) compared to those without eating disorders ($7,418) in the United States in 2016. Whilst day patient treatment is less costly than inpatient, a recent scoping review suggests that it is less cost-effective than outpatient treatment for those with EDs (44).

Although common intensive ED treatments, such as inpatient and day treatment, clearly have benefits for patients, including a focus on recovery, regular meal support, and improving physical health, they are also likely to bring additional challenges. For instance, inpatient treatment is associated with difficulties in maintaining relationships with friends and family, a sense of disconnection from real life (i.e. the potential for patients becoming institutionalised is high), and causes disruptions to education or work routines (45–47). In addition, the risk of relapse during the first months post-discharge is high (48–50). In contrast, day patient treatment allows patients to better maintain their relationship to the real world whilst receiving intensive treatment. However, just like inpatient treatment, attending a day patient programme and thus being at the hospital 3 to 7 days a week has the potential for disrupting education, employment and leisure activities (51). In addition, similar unintended iatrogenic effects as in inpatient treatment may be found in day programmes, in that strict schedules and routines around eating and predictable menus may produce an environment that fosters patients' inflexibility and reduces personal agency and self-efficacy (44). Moreover, outside urban areas with good transport connections, the daily commute to a day patient service can be challenging or impossible (e.g., 52,53). For example, the Mental Welfare Commission has reported that in Scotland, day patient services are geographically inequitable, leading to particular difficulties in accessing support in the community for those living in rural areas (53). Lastly, day patient treatment can also place significant burdens on patients' families, e.g. through disruptions to their daily routines and them having to carry increased responsibility for acutely unwell patients' physical and mental health (54,55). All in all, traditional institutionally based intensive treatments such as inpatient and day treatment are costly, have limited availability and are associated with a number of disadvantages for patients. Additionally, a recent report

For peer review only - http://bmjopen.bmj.com/site/about/guidelines.xhtml

For peer review only

by BEAT (56), the UK's leading ED charity for patients and carers, highlighted that patients and families see intensive community and home-based treatment modalities as more acceptable and empowering alternatives to traditional inpatient treatment. Therefore, alternative, and more flexible intensive treatment options that better align with patient preferences need to be explored to improve patient and carer outcomes and treatment experiences, manage increased demand for intensive treatments, and optimally employ scarce resources.

Intensive community (i.e., treatment, care and support offered within a patient's local environment in outpatient or community settings) and home-based (i.e., treatment, care and support offered within a patient's home) treatments are considered as alternatives to inpatient and day patient treatments. The benefits of these treatments have previously been explored for other severe mental disorders, such as psychosis and mood disorders, in response to the significant challenges associated with traditional institutional care (e.g. high treatment costs, long waiting periods, disruptions to daily life, institutionalisation). These treatments were designed to increase direct and easy access to care within an everyday life context (e.g., home, community centres and clinics) and minimise hospital (re-)admissions and days spent in inpatient units (57–59). Intensive community and home-based treatments are usually carried out by a multidisciplinary team and include several clinical contacts per week with flexibility in their provision, according to clinical need. Investigations of intensive community and home-based treatments have provided evidence for their effectiveness and usefulness for managing mental disorders (e.g., schizophrenia and other forms of psychosis, personality disorders) that previously would have been treated within an institutional setting. For instance, a naturalistic study on patients with acute mental disorders found that intensive home-based treatment is an effective and cost-effective alternative to inpatient treatment for reducing acute psychotic and depressive symptoms as well as clinical and functional impairment (60). Similarly, in a mixed psychiatric population intensive home-based treatment improved psychiatric symptoms, suicidality, general functioning, and caregiver burden (61). A randomised controlled trial of adolescents presenting with self-harm and admitted to inpatient services, compared inpatient treatment as usual to early discharge followed by intensive community care. Overall outcomes were similar in both groups, but patients allocated to intensive community care were significantly less likely to report multiple episodes of self-harm (62). Furthermore, these care models have been shown to be a valuable alternative to inpatient treatment through avoiding hospital admissions (63), thereby decreasing hospital days and readmission rates (62,64). Overall, the evidence suggests that these treatment modalities are cost-effective, and have been

For peer review only - http://bmjopen.bmj.com/site/about/guidelines.xhtml

to linked to positive clinical and social outcomes as well as rates of patient and carer satisfaction comparable to institutionally based intensive treatments (65–68). Additionally, the community-based nature of these treatment modalities may improve accessibility and circumvent some of the barriers to care outlined previously.

The possible advantages of intensive community and home-based approaches have been proposed for the treatment of EDs (69–71), however no attempt has yet been made to systematically review this area of research. We therefore aim to map literature on intensive community and home treatments for EDs through focusing on their conceptualisation, implementation and clinical outcomes. We further aim to identify knowledge gaps and provide directions for future research.

Research Questions

i. What is the extent of the available literature on intensive community and home-based treatments for EDs?
ii. How are intensive community- and home-based treatments for EDs conceptualised and implemented?
iii. What is the evidence on the efficacy, acceptability and cost-effectiveness of intensive community- and home-based treatments for EDs?

METHODS AND ANALYSIS

A scoping review will be conducted to map out the literature on intensive community and home-based treatments for ED. This study will follow the Preferred Reporting Items for Systematic Review and Meta-Analysis Protocol extension for Scoping Reviews checklist (PRISMA-ScR) (72) and the Joanna Briggs Institute (JBI) Reviewer's Manual (73).

Eligibility criteria

Population: This review will consider studies in patients with any type of ED diagnosis with any age and gender, including those with comorbid psychiatric diagnoses.

Concept: This review will consider any studies investigating intensive community and home-based treatments for EDs. We define intensive community treatment as any treatment offering

For peer review only - http://bmjopen.bmj.com/site/about/guidelines.xhtml

more than two therapeutic contacts per week, excluding mere physical monitoring contacts. This intensity cut-off was used as some outpatient therapies for EDs initially offer 2 sessions per week.

Context: This review will consider studies conducted in any geographical context or any community or home setting.

Types of evidence source: This review will consider studies with all types of research designs (e.g., randomised controlled trials, clinically controlled studies, quasi-experimental and case control studies, observational studies, case studies) and studies using quantitative, qualitative, or mixed methods. Ongoing clinical trials listed in trials registries will also be considered in order to explore further how intensive community and home treatments are being conceptualised and studied. Evidence from the grey literature will also be considered. Only studies published in English will be included.

Studies will be excluded if they (i) include mixed psychiatric populations, (ii) describe non-intensive treatments (i.e., offering fewer than three therapeutic sessions per week), and (iii) describe institution/hospital-based treatments (i.e., inpatient and day patient).

Search strategy

The literature search will be conducted on four main databases: PubMed, PsycInfo, MEDLINE, Web of Science. Sources of unpublished studies and of grey literature will include Scopus, Google Scholar, US ClinicalTrials.gov, WHO International clinical trials registry platform search portal, ISRCTN Registry, and the European Union Clinical Trials Register. Finally, we will conduct a reference list search on the final included studies to identify any additional relevant studies.

A limited initial search was conducted in PubMed by Bİ in February 2022, to identify relevant keywords and develop a search strategy. Then, a preliminary search strategy was developed by Bİ and MP and reviewed by US. The search strategy will be tailored to each database by Bİ and MP, and the search will be conducted on studies published until 1st February 2023. The search terms will include variations of “feeding and eating disorders”, “community treatment”, “intensive outpatient”, and “home treatment”. A pilot search conducted on PubMed, PsycInfo, MEDLINE, Web of Science on 01/07/2022 are presented in the Supplemental material.

For peer review only - http://bmjopen.bmj.com/site/about/guidelines.xhtml

Study selection

After completing the searches, references will be uploaded to Endnote citation management software, and duplicates will be removed. Titles and abstracts will be reviewed and remaining full-text citations will be screened for eligibility by Bİ and MP independently. In the case of any discrepancies, the reviewers will meet to discuss relevant studies. If the two reviewers cannot reach an agreement on the eligibility of a study, this disagreement will be resolved through consultation with US. Rayyan software will be used in this process.

Data charting

The data charting form developed by the present authors will be used to extract the key information relevant to study aims. The draft data charting form is presented in Table 1. This form might be updated during the final review process in order to ensure all relevant data are accurately captured.

The data will be charted by Bİ and MP independently for each of the studies, to assess if they meet inclusion criteria. If any disagreement arises, the reviewers will discuss to reach a consensus. US will be consulted in the case of uncertainty or unsolved disagreement.

Table 1 Draft Data Charting From

Data Item	Data
Author(s)	
Publication Year	
Location & Country	
Study Design	
Study Population and Sample Size	
Intervention Type	
Intervention Length & Dosage	
Underlying Theoretical Model for the Intervention	
Intervention Delivery Mode	
Professional(s) Delivering the Intervention	
Involvement of Carers in the Intervention	
Admission & Discharge Criteria (if available)	

For peer review only - http://bmjopen.bmj.com/site/about/guidelines.xhtml

Main findings	

Data analysis and synthesis

The detailed information regarding the selection procedure will be presented in a PRISMA Flow Diagram. A summary of the studies using the data charting form will be provided in a table with an accompanying narrative description relating to the research aims of this review. Where relevant, common and distinctive characteristics of the treatment modalities will be identified. The main findings will be considered in terms of a range of physical, psychological, social, and functional outcomes to provide a comprehensive and holistic overview of the clinical outcomes. The strength of the evidence will be examined using the JBI Levels of Evidence for Effectiveness: Level 1 (high) - experimental designs, Level 2 - quasi-experimental designs, Level 3 - observational – analytic designs, Level 4 - observational–descriptive and Level 5 (low) - Expert Opinion. To guide clinical applications, future research and policy makers, knowledge gaps will be highlighted.

Patient and public involvement

No patients or members of the public were involved in the development of this protocol or will be involved in the final scoping review. Nevertheless, including qualitative studies should give a valuable insight into patient and carer perceptions and experiences with intensive community and home treatments.

ETHICS AND DISSEMINATION

Ethical approval will not be required for this review as the data will be obtained from publicly available sources. Dissemination of the review's findings will be performed through peer-reviewed publication and through conference presentations. Additionally, we will use social media (i.e., Twitter and Instagram) to reach the wider public.

Summary and Conclusion

To our knowledge, this will be the first review to map out the literature on non-institutional (i.e. community and home based) intensive treatments for ED. The findings will provide an overview on the extent, characteristics, clinical outcomes, and cost-effectiveness of these non-institutionally based intensive treatments. Additionally, this review will provide insights into patient and carer experiences with these interventions. We expect that this review will suggest

For peer review only - http://bmjopen.bmj.com/site/about/guidelines.xhtml

future directions for designing new intensive treatment services or improving the existing ones, as well as for developing research priorities and policies.

Authors' contributions: US conceived of the idea for this scoping review. Bİ developed the research questions, search strategy, data charting tool and data analysis plan with support from MP. Bİ and MP drafted the manuscript. US supervised the protocol development and critically reviewed the manuscript. All authors have read and approved final version of the manuscript.

Funding: US receives salary support from the National Institute for Health Research (NIHR) Biomedical Research Centre for Mental Health, South London and Maudsley NHS Foundation Trust and Institute of Psychiatry, Psychology and Neuroscience, King's College London.

Competing interests: None declared.

Patient consent for publication: Not required.

For peer review only

REFERENCES

1. Schaumberg K, Welch E, Breithaupt L, Hübel C, Baker JH, Munn-Chernoff MA, et al. The Science Behind the Academy for Eating Disorders' Nine Truths About Eating Disorders. Eur Eat Disord Rev. 2017 Nov;25(6):432–50.
2. van Hoeken D, Hoek HW. Review of the burden of eating disorders: mortality, disability, costs, quality of life, and family burden. Curr Opin Psychiatry. 2020;33(6).
3. Treasure J, Duarte TA, Schmidt U. Eating disorders. Lancet (London, England). 2020 Mar;395(10227):899–911.
4. Keski-Rahkonen A, Mustelin L. Epidemiology of eating disorders in Europe: prevalence, incidence, comorbidity, course, consequences, and risk factors. Curr Opin Psychiatry. 2016/09/24. 2016;29(6):340–5.
5. Portzky G, van Heeringen K, Vervaet M. Attempted suicide in patients with eating disorders. Vol. 35, Crisis: The Journal of Crisis Intervention and Suicide Prevention. Portzky, Gwendolyn: Unit for Suicide Research–Ghent University, University Hospital Ghent, 1K12F, De Pintelaan 185, Ghent, Belgium, 9000, gwendolyn.portzky@ugent.be: Hogrefe Publishing; 2014. p. 378–87.
6. Giel KE, Bulik CM, Fernandez-Aranda F, Hay P, Keski-Rahkonen A, Schag K, et al. Binge eating disorder. Nat Rev Dis Prim. 2022;8(1):16.
7. Mehler PS, Rylander M. Bulimia Nervosa - medical complications. J Eat Disord. 2015/04/14. 2015;3:12.
8. Treasure J, Zipfel S, Micali N, Wade T, Stice E, Claudino A, et al. Anorexia nervosa. Nat Rev Dis Prim. 2015;1(1):15075.
9. Fichter MM, Quadflieg N. Mortality in eating disorders - results of a large prospective clinical longitudinal study. Int J Eat Disord. 2016 Apr;49(4):391–401.
10. Arcelus J, Mitchell AJ, Wales J, Nielsen S. Mortality Rates in Patients With Anorexia Nervosa and Other Eating Disorders: A Meta-analysis of 36 Studies. Arch Gen Psychiatry. 2011 Jul;68(7):724–31.
11. Wonderlich S, Mitchell JE, Crosby RD, Myers TC, Kadlec K, LaHaise K, et al. Minimizing and treating chronicity in the eating disorders: A clinical overview. Int J Eat Disord. 2012 May;45(4):467–75.
12. Linardon J, Messer M, Rodgers RF, Fuller-Tyszkiewicz M. A systematic scoping review of research on COVID-19 impacts on eating disorders: A critical appraisal of the evidence and recommendations for the field. Int J Eat Disord. 2022 Jan;55(1):3–38.
13. de Graaf R, ten Have M, van Gool C, van Dorsselaer S. Prevalence of mental disorders

For peer review only - http://bmjopen.bmj.com/site/about/guidelines.xhtml

and trends from 1996 to 2009. Results from the Netherlands Mental Health Survey and Incidence Study-2 . Soc Psychiatry Psychiatr Epidemiol. 2012;47(2):203–13.

14. Baxter AJ, Scott KM, Ferrari AJ, Norman RE, Vos T, Whiteford HA. Challenging the myth of an “epidemic” of common mental disorders: Trends in the global prevalence of anxiety and depression between 1990 and 2010. Depress Anxiety. 2014 Jun;31(6):506–16.

15. Green BH, Griffiths EC. Hospital admission and community treatment of mental disorders in England from 1998 to 2012. Gen Hosp Psychiatry. 2014;36(4):442–8.

16. Degli Esposti M, Ziauddeen H, Bowes L, Reeves A, Chekroud AM, Humphreys DK, et al. Trends in inpatient care for psychiatric disorders in NHS hospitals across England, 1998/99–2019/20: an observational time series analysis. Soc Psychiatry Psychiatr Epidemiol. 2021;

17. Schlegl S, Maier J, Meule A, Voderholzer U. Eating disorders in times of the COVID-19 pandemic—Results from an online survey of patients with anorexia nervosa. Int J Eat Disord. 2020 Nov;53(11):1791–800.

18. Giel KE, Schurr M, Zipfel S, Junne F, Schag K. Eating behaviour and symptom trajectories in patients with a history of binge eating disorder during COVID-19 pandemic. Eur Eat Disord Rev. 2021 Jul;29(4):657–62.

19. Asch DA, Buresh J, Allison KC, Islam N, Sheils NE, Doshi JA, et al. Trends in US Patients Receiving Care for Eating Disorders and Other Common Behavioral Health Conditions Before and During the COVID-19 Pandemic. JAMA Netw Open. 2021 Nov;4(11):e2134913–e2134913.

20. Lin JA, Hartman-Munick SM, Kells MR, Milliren CE, Slater WA, Woods ER, et al. The Impact of the COVID-19 Pandemic on the Number of Adolescents/Young Adults Seeking Eating Disorder-Related Care. J Adolesc Heal. 2021;69(4):660–3.

21. Toulany A, Kurdyak P, Guttmann A, Stukel TA, Fu L, Strauss R, et al. Acute Care Visits for Eating Disorders Among Children and Adolescents After the Onset of the COVID-19 Pandemic. J Adolesc Heal. 2022;70(1):42–7.

22. NICE. Eating disorders: Recognition and treatment. NICE guideline. London: National Institute for Health and Care Excellence; 2017.

23. American Psychiatric Association. Treatment of patients with eating disorders,third edition. Am J Psychiatry. 2006 Jul;163(7 Suppl):4–54.

24. Heruc G, Hurst K, Casey A, Fleming K, Freeman J, Fursland A, et al. ANZAED eating disorder treatment principles and general clinical practice and training standards. J Eat

For peer review only - http://bmjopen.bmj.com/site/about/guidelines.xhtml

Disord. 2020;8(1):63.

25. Madden S, Hay P, Touyz S. Systematic review of evidence for different treatment settings in anorexia nervosa. World J psychiatry. 2015 Mar;5(1):147–53.
26. Morris J, Simpson A V, Voy SJ. Length of stay of inpatients with eating disorders. Vol. 22, Clinical Psychology & Psychotherapy. Morris, Jane: Eden Unit, Royal Cornhill Hospital, Aberdeen, Scotland, AB25 2ZH, janemorris1@nhs.net: John Wiley & Sons; 2015. p. 45–53.
27. Cruz AM, Gonçalves-Pinho M, Santos JV, Coutinho F, Brandão I, Freitas A. Eating disorders—Related hospitalizations in Portugal: A nationwide study from 2000 to 2014. Int J Eat Disord. 2018 Oct;51(10):1201–6.
28. Kan C, Hawkings Y-R, Cribben H, Treasure J. Length of stay for anorexia nervosa: Systematic review and meta-analysis. Eur Eat Disord Rev. 2021 May;29(3):371–92.
29. Zipfel S, Reas DL, Thornton C, Olmsted MP, Williamson DA, Gerlinghoff M, et al. Day hospitalization programs for eating disorders: A systematic review of the literature. Int J Eat Disord. 2002 Mar;31(2):105–17.
30. Brown TA, Cusack A, Anderson LK, Trim J, Nakamura T, Trunko ME, et al. Efficacy of a partial hospital programme for adults with eating disorders. Eur Eat Disord Rev. 2018 May;26(3):241–52.
31. Abbate-Daga G, Gramaglia C, Preda S, Comba E, Brustolin A, Fassino S. Day hospital programmes for eating disorders: A review of the similarities, differences and goals. Eat Weight Disord - Stud Anorexia, Bulim Obes. 2009;14(2):e31–41.
32. Friedman K, Ramirez AL, Murray SB, Anderson LK, Cusack A, Boutelle KN, et al. A Narrative Review of Outcome Studies for Residential and Partial Hospital-based Treatment of Eating Disorders. Eur Eat Disord Rev. 2016 Jul;24(4):263–76.
33. Makhzoumi SH, Schreyer CC, Hansen JL, Laddaran LA, Redgrave GW, Guarda AS. Hospital course of underweight youth with ARFID treated with a meal-based behavioral protocol in an inpatient-partial hospitalization program for eating disorders. Int J Eat Disord. 2019 Apr;52(4):428–34.
34. Baudinet J, Simic M. Adolescent Eating Disorder Day Programme Treatment Models and Outcomes: A Systematic Scoping Review. Front psychiatry. 2021;12:652604.
35. Guarda AS, Schreyer CC, Fischer LK, Hansen JL, Coughlin JW, Kaminsky MJ, et al. Intensive treatment for adults with anorexia nervosa: The cost of weight restoration. Int J Eat Disord. 2017 Mar;50(3):302–6.
36. Williamson DA, Thaw JM, Varnado-Sullivan PJ. Cost-effectiveness analysis of a

For peer review only - http://bmjopen.bmj.com/site/about/guidelines.xhtml

hospital-based cognitive-behavioral treatment program for eating disorders. Behav Ther. 2001;32(3):459–77.

37. Byford S, Barrett B, Roberts C, Clark A, Edwards V, Smethurst N, et al. Economic evaluation of a randomised controlled trial for anorexia nervosa in adolescents. Br J Psychiatry. 2018/01/02. 2007;191(5):436–40.

38. Haas L, Stargardt T, Schreyoegg J, Schlösser R, Danzer G, Klapp BF. Inpatient costs and predictors of costs in the psychosomatic treatment of anorexia nervosa. Int J Eat Disord. 2012 Mar;45(2):214–21.

39. Toulany A, Wong M, Katzman DK, Akseer N, Steinegger C, Hancock-Howard RL, et al. Cost analysis of inpatient treatment of anorexia nervosa in adolescents: hospital and caregiver perspectives. C Open. 2015 Apr;3(2):E192 LP-E197.

40. Stuhldreher N, Wild B, König H-H, Konnopka A, Zipfel S, Herzog W. Determinants of direct and indirect costs in anorexia nervosa. Int J Eat Disord. 2015 Jan;48(1):139–46.

41. Ágh T, Kovács G, Supina D, Pawaskar M, Herman BK, Vokó Z, et al. A systematic review of the health-related quality of life and economic burdens of anorexia nervosa, bulimia nervosa, and binge eating disorder. Eat Weight Disord - Stud Anorexia, Bulim Obes. 2016;21(3):353–64.

42. Samnaliev M, Noh HL, Sonneville KR, Austin SB. The economic burden of eating disorders and related mental health comorbidities: An exploratory analysis using the U.S. Medical Expenditures Panel Survey. Prev Med Reports. 2015;2:32–4.

43. Presskreischer R, Steinglass JE, Anderson KE. Eating disorders in the U.S. Medicare population. Int J Eat Disord [Internet]. 2022 Mar 1;55(3):362–71. Available from: https://doi.org/10.1002/eat.23676

44. Ali SI, Bodnar E, Gamberg S, Bartel SJ, Waller G, Nunes A, et al. The costs and benefits of intensive day treatment programs and outpatient treatments for eating disorders: An idea worth researching. Int J Eat Disord. 2021 Jul;54(7):1099–105.

45. Offord A, Turner H, Cooper M. Adolescent inpatient treatment for anorexia nervosa: a qualitative study exploring young adults' retrospective views of treatment and discharge. Eur Eat Disord Rev. 2006 Nov;14(6):377–87.

46. Bezance J, Holliday J. Adolescents with Anorexia Nervosa Have Their Say: A Review of Qualitative Studies on Treatment and Recovery from Anorexia Nervosa. Eur Eat Disord Rev. 2013 Sep;21(5):352–60.

47. Elran-Barak R, Barak A, Cohen-Ashkenazi S, Schifter T. Barriers to hospital

For peer review only - http://bmjopen.bmj.com/site/about/guidelines.xhtml

admission among people with eating disorders: A qualitative internet-based study. Int J Ment Health Nurs. 2018 Dec;27(6):1784–92.

48. Richard M, Bauer S, Kordy H. Relapse in anorexia and bulimia nervosa—a 2.5-year follow-up study. Eur Eat Disord Rev. 2005 May;13(3):180–90.

49. Walsh BT, Xu T, Wang Y, Attia E, Kaplan AS. Time Course of Relapse Following Acute Treatment for Anorexia Nervosa. Am J Psychiatry. 2021 Jun;178(9):848–53.

50. Khalsa SS, Portnoff LC, McCurdy-McKinnon D, Feusner JD. What happens after treatment? A systematic review of relapse, remission, and recovery in anorexia nervosa. J Eat Disord. 2017;5(1):20.

51. Matthews K, Gordon L, van Beusekom J, Sheffield J, Painter E, Bellair R, et al. A day treatment program for adults with eating disorders: staff and patient experiences in implementation. J Eat Disord. 2019;7(1):21.

52. Nikendei C, Haitz M, Huber J, Ehrenthal JC, Herzog W, Schauenburg H, et al. Day clinic and inpatient psychotherapy of depression (DIP-D): qualitative results from a randomized controlled study. Int J Ment Health Syst. 2016;10(1):41.

53. Mental Welfare Commission. Mental Welfare Commission for Scotland. Mapping of Eating Disorder Services across Scotland. 2020.

54. Fox JR, Dean M, Whittlesea A. The Experience of Caring For or Living with an Individual with an Eating Disorder: A Meta-Synthesis of Qualitative Studies. Clin Psychol Psychother. 2017 Jan;24(1):103–25.

55. Robinson I, Stoyel H, Robinson P. "If she had broken her leg she would not have waited in agony for 9 months": Caregiver's experiences of eating disorder treatment. Eur Eat Disord Rev. 2020 Nov;28(6):750–65.

56. Beat. Intensive day- and home-based treatment for eating disorders: an effective and less expensive alternative to inpatient care. 2019.

57. Burns T. Community mental health teams. Psychiatry. 2004;3(9):11–4.

58. Johnson S. Crisis resolution and intensive home treatment teams. Psychiatry. 2004;3(9):22–5.

59. Johnson S. Crisis resolution and home treatment teams: an evolving model. Adv Psychiatr Treat. 2018/01/02. 2013;19(2):115–23.

60. Kilian R, Becker T, Frasch K. Effectiveness and cost-effectiveness of home treatment compared with inpatient care for patients with acute mental disorders in a rural catchment area in Germany. Neurol Psychiatry Brain Res. 2016;22(2):81–6.

61. Gotink RA, Bergsma G, Hoogwegt MT, Mulder CL, Van Gool AR. Intensive home

For peer review only - http://bmjopen.bmj.com/site/about/guidelines.xhtml

treatment: een eerste observationele evaluatie. Tijdschr Psychiatr. 2017;59(11):713–9.

62. Ougrin D, Corrigall R, Stahl D, Poole J, Zundel T, Wait M, et al. Supported discharge service versus inpatient care evaluation (SITE): a randomised controlled trial comparing effectiveness of an intensive community care service versus inpatient treatment as usual for adolescents with severe psychiatric disorders: self-h. Eur Child Adolesc Psychiatry. 2021;30(9):1427–36.

63. Huang HCH, Taylor M, Carmichael A. The outcomes of home treatment for schizophrenia. BJPsych Bull. 2018/08/06. 2018 Dec;42(6):238–42.

64. Mötteli S, Schori D, Schmidt H, Seifritz E, Jäger M. Utilization and Effectiveness of Home Treatment for People With Acute Severe Mental Illness: A Propensity-Score Matching Analysis of 19 Months of Observation . Vol. 9, Frontiers in Psychiatry . 2018.

65. Boege I, Corpus N, Schepker R, Kilian R, Fegert JM. Cost-effectiveness of intensive home treatment enhanced by inpatient treatment elements in child and adolescent psychiatry in Germany: A randomised trial. Eur Psychiatry. 2020/04/15. 2015;30(5):583–9.

66. Stulz N, Wyder L, Maeck L, Hilpert M, Lerzer H, Zander E, et al. Home treatment for acute mental healthcare: randomised controlled trial. Br J Psychiatry. 2019/03/13. 2020;216(6):323–30.

67. Graf D, Lerch S, Böhnke U, Reichl C, Kindler J, Koenig J, et al. Treatment outcome of an intensive psychiatric home treatment for children and adolescents: a non-randomized controlled pilot evaluation. Eur Child Adolesc Psychiatry. 2021;

68. Kwok KHR, Yuan SNV, Ougrin D. Review: Alternatives to inpatient care for children and adolescents with mental health disorders. Child Adolesc Ment Health. 2016 Feb;21(1):3–10.

69. Herpertz-Dahlmann B. Intensive Treatments in Adolescent Anorexia Nervosa. Vol. 13, Nutrients . 2021.

70. Munro C, Thomson V, Corr J, Randell L, Davies JE, Gittoes C, et al. A new service model for the treatment of severe anorexia nervosa in the community: the Anorexia Nervosa Intensive Treatment Team. Psychiatr Bull. 2018/01/02. 2014;38(5):220–5.

71. Pauli D, Flütsch N, Hilti N, Schräer C, Soumana M, Häberling I, et al. Home treatment as an add-on to family-based treatment in adolescents with anorexia nervosa: A pilot study. Eur Eat Disord Rev [Internet]. 2022 Mar 1;30(2):168–77. Available from: https://doi.org/10.1002/erv.2882

For peer review only - http://bmjopen.bmj.com/site/about/guidelines.xhtml

72. Tricco AC, Lillie E, Zarin W, O'Brien KK, Colquhoun H, Levac D, et al. PRISMA Extension for Scoping Reviews (PRISMA-ScR): Checklist and Explanation. Ann Intern Med. 2018 Sep;169(7):467–73.

73. Peters MDJ, Godfrey C, McInerney P, Munn Z, Tricco AC, Khalil H. JBI Manual for Evidence Synthesis, JBI, 2020. In: Aromataris E, Munn Z, editors. 2020.

For peer review only

For peer review only - http://bmjopen.bmj.com/site/about/guidelines.xhtml

Supplemental material

This document provides details of the pilot search conducted on the main databases on 01/07/2022.

Pilot search on PubMed

	Query	Results
**#1**	"Home Care Services"[Mesh] OR "Home Care Services, Hospital-Based"[Mesh] OR "Community Mental Health Services"[Mesh] OR "Community Mental Health Centers"[Mesh]	71545
**#2**	"community care*"[Title/Abstract] OR "community treat*"[Title/Abstract] OR "intensive community*"[Title/Abstract] OR "intensive outpatient*"[Title/Abstract] OR "home care*"[Title/Abstract] OR "home treat*"[Title/Abstract] OR "home feed*"[Title/Abstract]	31346
**#3**	"Anorexia Nervosa"[MeSH Terms] OR "Anorexia"[MeSH Terms] OR "Avoidant Restrictive Food Intake Disorder"[MeSH Terms] OR "Binge-Eating Disorder"[MeSH Terms] OR "Bulimia Nervosa"[MeSH Terms] OR "Bulimia"[MeSH Terms] OR"Feeding and Eating Disorders"[MeSH Terms]	41989
**#4**	"eating disorder*"[Title/Abstract] OR "anorex*"[Title/Abstract] OR "bulimi*"[Title/Abstract] OR "binge eating*"[Title/Abstract] OR "Avoidant Restrictive Food Intake Disorder" OR"ARFID"[Title/Abstract]	57264
**#5**	#1 OR #2	88293
**#6**	#3 OR #4	68288
**#7**	#5 AND #6	238

For peer review only

For peer review only - http://bmjopen.bmj.com/site/about/guidelines.xhtml

Pilot search on APA PsycInfo

	Query	Results
**#1**	exp Community Mental Health Services/ or exp Community Mental Health Centers/	10681
**#2**	exp Home Care/	7190
**#3**	(community care* or community treat* or intensive community* or intensive outpatient*).mp.	6807
**#4**	(home adj3 care*).mp.	19244
**#5**	(home adj3 treat*).mp.	1927
**#6**	(home adj3 feed*).mp.	178
**#7**	exp Eating Disorders/	33619
**#8**	(Feeding and Eating Disorder*).mp.	9239
**#9**	(anorex* or bulimi* or binge eat* or Avoidant Restrictive Food Intake Disorder or ARFID).mp.	29770
**#10**	(eating adj3 disorder*).mp.	35442
**#11**	#1 or #2 or #3 or #4 or #5 or #6	36110
**#12**	#7 or #8 or #9 or #10	49186
**#13**	#11 and #12	204

For peer review only

Pilot search on Ovid MEDLINE

	Query	Results
**#1**	exp Community Mental Health Centers/ or exp Community Mental Health Services/	21939
**#2**	exp Home Care Services/	50063
**#3**	(community care* or community treat* or intensive community* or intensive outpatient*).mp. [mp=title, abstract, original title, name of substance word, subject heading word, floating sub-heading word, keyword heading word, organism supplementary concept word, protocol supplementary concept word, rare disease supplementary concept word, unique identifier, synonyms]	8610
**#4**	(home adj3 care*).mp. [mp=title, abstract, original title, name of substance word, subject heading word, floating sub-heading word, keyword heading word, organism supplementary concept word, protocol supplementary concept word, rare disease supplementary concept word, unique identifier, synonyms]	65503
**#5**	(home adj3 treat*).mp. [mp=title, abstract, original title, name of substance word, subject heading word, floating sub-heading word, keyword heading word, organism supplementary concept word, protocol supplementary concept word, rare disease supplementary concept word, unique identifier, synonyms]	5993
**#6**	(home adj3 feed*).mp. [mp=title, abstract, original title, name of substance word, subject heading word, floating sub-heading word, keyword heading word, organism supplementary concept word, protocol supplementary concept word, rare disease supplementary concept word, unique identifier, synonyms]	670
**#7**	exp "Feeding and Eating Disorders"/	34639
**#8**	(anorex* or bulimi* or binge eating* or Avoidant Restrictive Food Intake Disorder or ARFID).mp. [mp=title, abstract, original title, name of substance word, subject heading word, floating sub-heading word, keyword heading word, organism supplementary concept word, protocol supplementary concept word, rare disease supplementary concept word, unique identifier, synonyms]	50023
**#9**	(eating adj3 disorder*).mp. [mp=title, abstract, original title, name of substance word, subject heading word, floating sub-heading word, keyword heading word, organism supplementary concept word, protocol supplementary concept word, rare disease supplementary concept word, unique identifier, synonyms]	32939
**#10**	#1 or #2 or #3 or #4 or #5 or #6	107933
**#11**	#7 or #8 or #9	69172
**#12**	#10 and #11	293

For peer review only - http://bmjopen.bmj.com/site/about/guidelines.xhtml

Pilot search on Web of Science

	Query	Results
#1	TS=("eating disorder*" OR "anorex*" OR "bulimi*" OR "binge eat*" OR "Avoidant Restrictive Food Intake Disorder" OR "ARFID")	70790
#2	TS=((community NEAR/3 "care*") OR (community NEAR/3 "treat*") OR (intensive NEAR/3 "community*") OR (intensive NEAR/3 "outpatient*") OR (home NEAR/3 "care*") OR (home NEAR/3 "treat*") OR (home NEAR/3 " feed*"))	99666
#3	#1 AND #2	288

For peer review only

Preferred Reporting Items for Systematic reviews and Meta-Analyses extension for Scoping Reviews (PRISMA-ScR) Checklist

SECTION	ITEM	PRISMA-ScR CHECKLIST ITEM	REPORTED ON PAGE #
**TITLE**			
Title	1	Identify the report as a scoping review.	1
**ABSTRACT**			
Structured summary	2	Provide a structured summary that includes (as applicable): background, objectives, eligibility criteria, sources of evidence, charting methods, results, and conclusions that relate to the review questions and objectives.	2
**INTRODUCTION**			
Rationale	3	Describe the rationale for the review in the context of what is already known. Explain why the review questions/objectives lend themselves to a scoping review approach.	4 to 8
Objectives	4	Provide an explicit statement of the questions and objectives being addressed with reference to their key elements (e.g., population or participants, concepts, and context) or other relevant key elements used to conceptualize the review questions and/or objectives.	8
**METHODS**			
Protocol and registration	5	Indicate whether a review protocol exists; state if and where it can be accessed (e.g., a Web address); and if available, provide registration information, including the registration number.	N/A
Eligibility criteria	6	Specify characteristics of the sources of evidence used as eligibility criteria (e.g., years considered, language, and publication status), and provide a rationale.	8 & 9
Information sources*	7	Describe all information sources in the search (e.g., databases with dates of coverage and contact with authors to identify additional sources), as well as the date the most recent search was executed.	9
Search	8	Present the full electronic search strategy for at least 1 database, including any limits used, such that it could be repeated.	Supplement
Selection of sources of evidence†	9	State the process for selecting sources of evidence (i.e., screening and eligibility) included in the scoping review.	9
Data charting process‡	10	Describe the methods of charting data from the included sources of evidence (e.g., calibrated forms or forms that have been tested by the team before their use, and whether data charting was done independently or in duplicate) and any processes for obtaining and confirming data from investigators.	10&11
Data items	11	List and define all variables for which data were sought and any assumptions and simplifications made.	N/A
Critical appraisal of individual sources of evidence§	12	If done, provide a rationale for conducting a critical appraisal of included sources of evidence; describe the methods used and how this information was used in any data synthesis (if appropriate).	N/A

For peer review only - http://bmjopen.bmj.com/site/about/guidelines.xhtml

SECTION	ITEM	PRISMA-ScR CHECKLIST ITEM	REPORTED ON PAGE #
Synthesis of results	13	Describe the methods of handling and summarizing the data that were charted.	11
**RESULTS**			
Selection of sources of evidence	14	Give numbers of sources of evidence screened, assessed for eligibility, and included in the review, with reasons for exclusions at each stage, ideally using a flow diagram.	N/A
Characteristics of sources of evidence	15	For each source of evidence, present characteristics for which data were charted and provide the citations.	N/A
Critical appraisal within sources of evidence	16	If done, present data on critical appraisal of included sources of evidence (see item 12).	N/A
Results of individual sources of evidence	17	For each included source of evidence, present the relevant data that were charted that relate to the review questions and objectives.	N/A
Synthesis of results	18	Summarize and/or present the charting results as they relate to the review questions and objectives.	N/A
**DISCUSSION**			
Summary of evidence	19	Summarize the main results (including an overview of concepts, themes, and types of evidence available), link to the review questions and objectives, and consider the relevance to key groups.	N/A
Limitations	20	Discuss the limitations of the scoping review process.	N/A
Conclusions	21	Provide a general interpretation of the results with respect to the review questions and objectives, as well as potential implications and/or next steps.	N/A
**FUNDING**			
Funding	22	Describe sources of funding for the included sources of evidence, as well as sources of funding for the scoping review. Describe the role of the funders of the scoping review.	12

JBI = Joanna Briggs Institute; PRISMA-ScR = Preferred Reporting Items for Systematic reviews and Meta-Analyses extension for Scoping Reviews.

* Where *sources of evidence* (see second footnote) are compiled from, such as bibliographic databases, social media platforms, and Web sites.

† A more inclusive/heterogeneous term used to account for the different types of evidence or data sources (e.g., quantitative and/or qualitative research, expert opinion, and policy documents) that may be eligible in a scoping review as opposed to only studies. This is not to be confused with *information sources* (see first footnote).

‡ The frameworks by Arksey and O'Malley (6) and Levac and colleagues (7) and the JBI guidance (4, 5) refer to the process of data extraction in a scoping review as data charting.

§ The process of systematically examining research evidence to assess its validity, results, and relevance before using it to inform a decision. This term is used for items 12 and 19 instead of "risk of bias" (which is more applicable to systematic reviews of interventions) to include and acknowledge the various sources of evidence that may be used in a scoping review (e.g., quantitative and/or qualitative research, expert opinion, and policy document).

From: Tricco AC, Lillie E, Zarin W, O'Brien KK, Colquhoun H, Levac D, et al. PRISMA Extension for Scoping Reviews (PRISMAScR): Checklist and Explanation. Ann Intern Med. 2018;169:467–473. doi: 10.7326/M18-0850.

For peer review only - http://bmjopen.bmj.com/site/about/guidelines.xhtml
